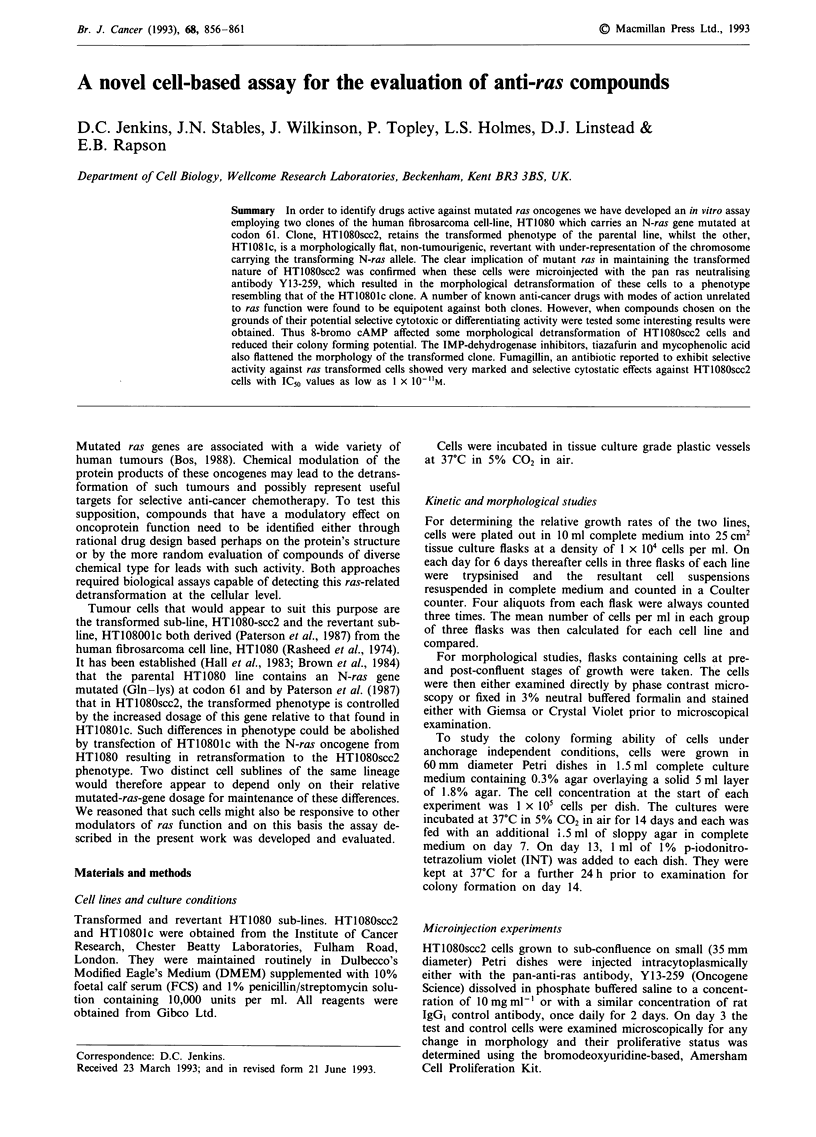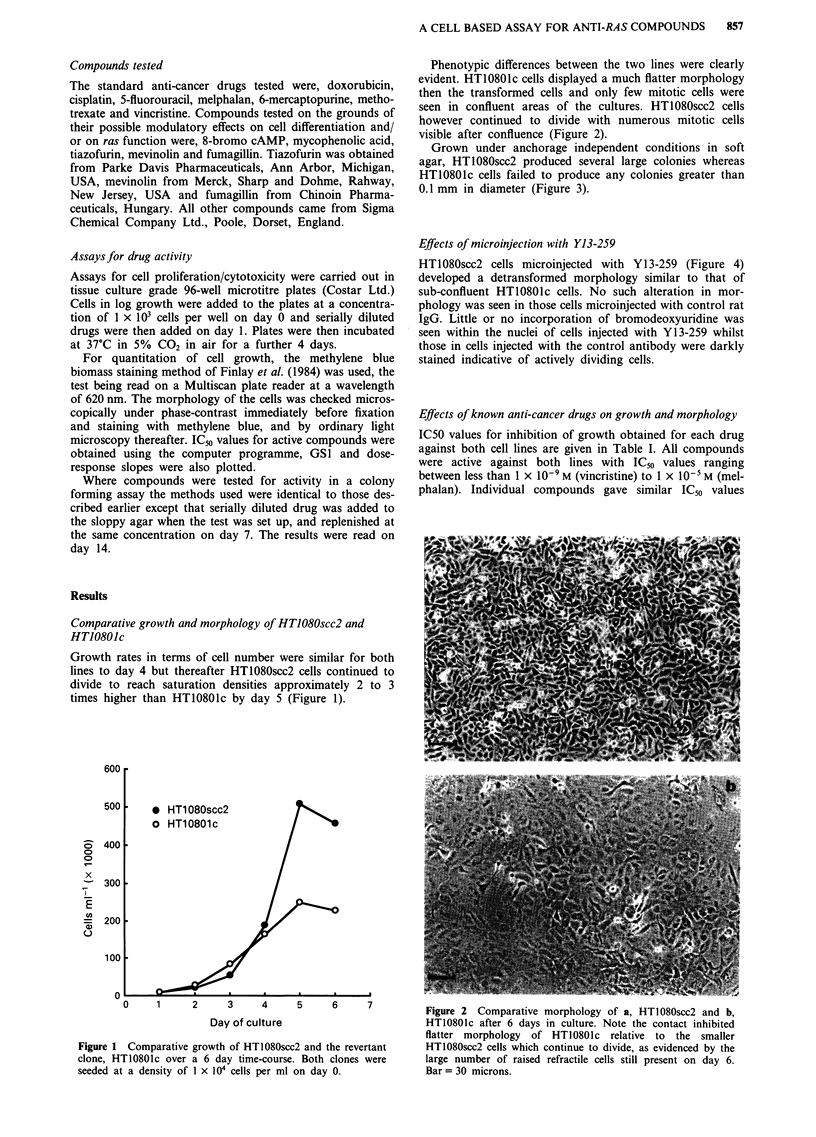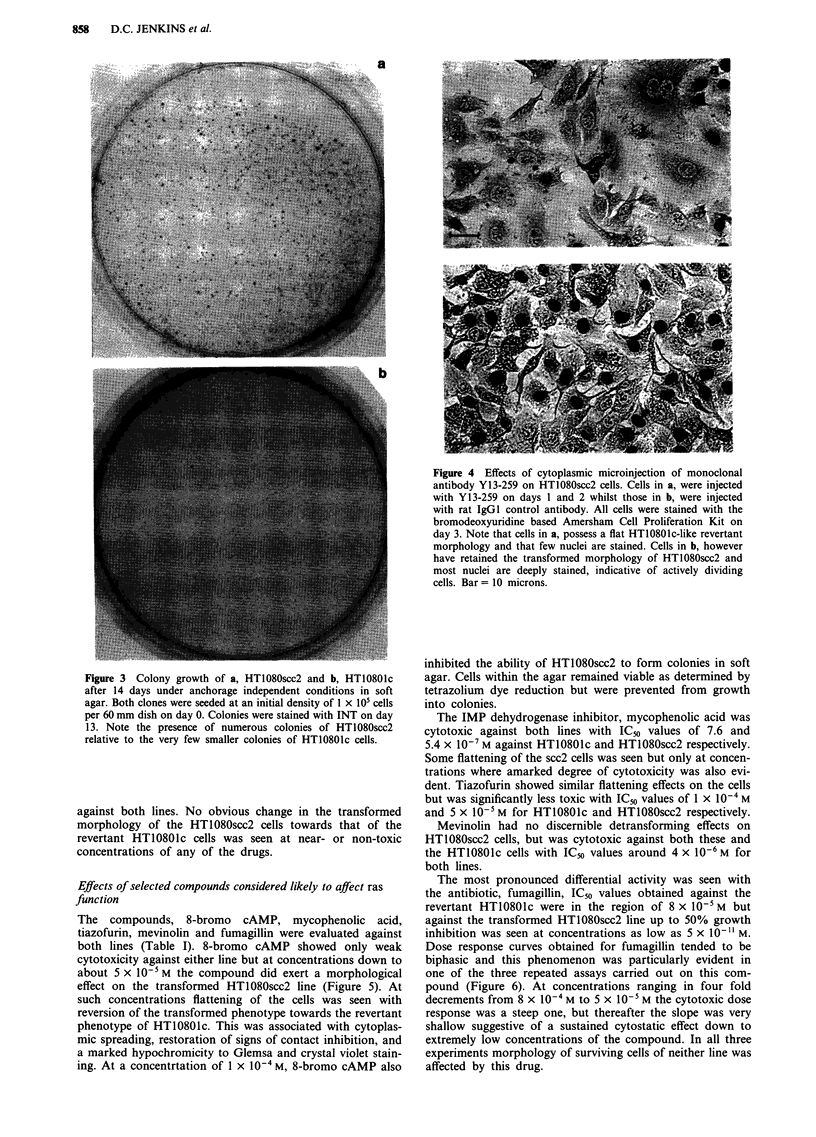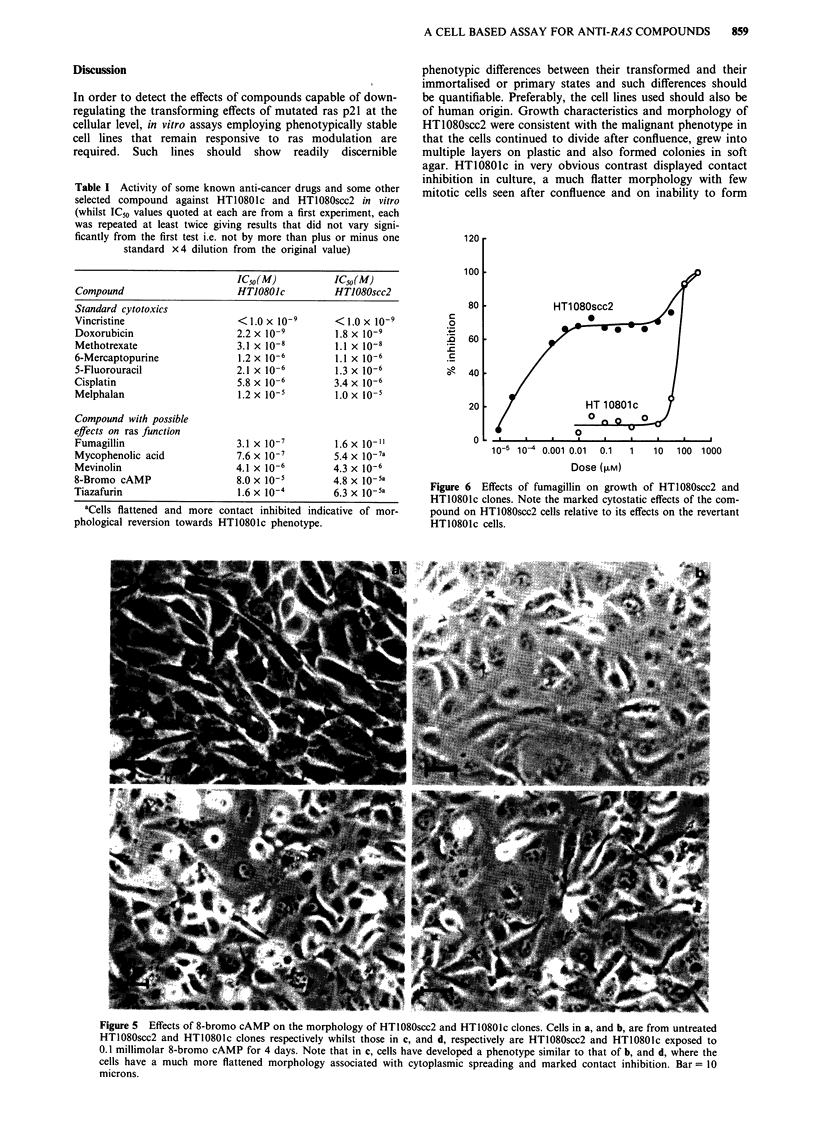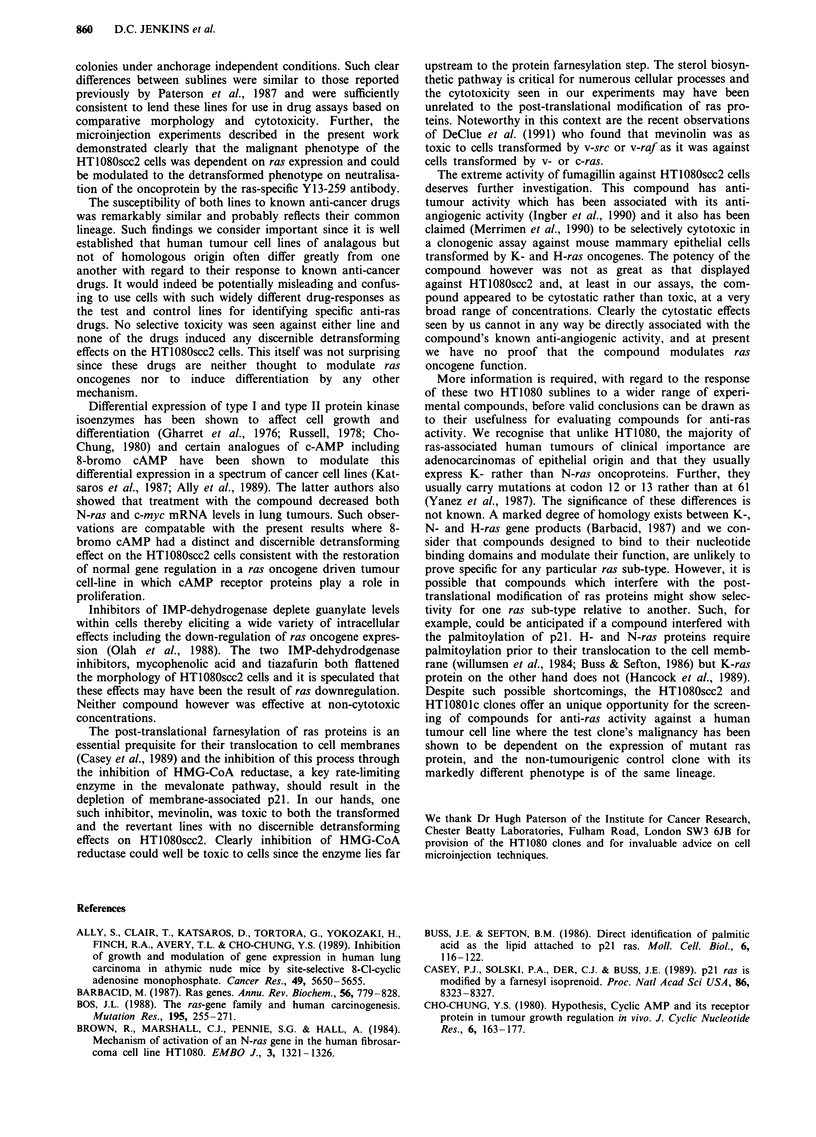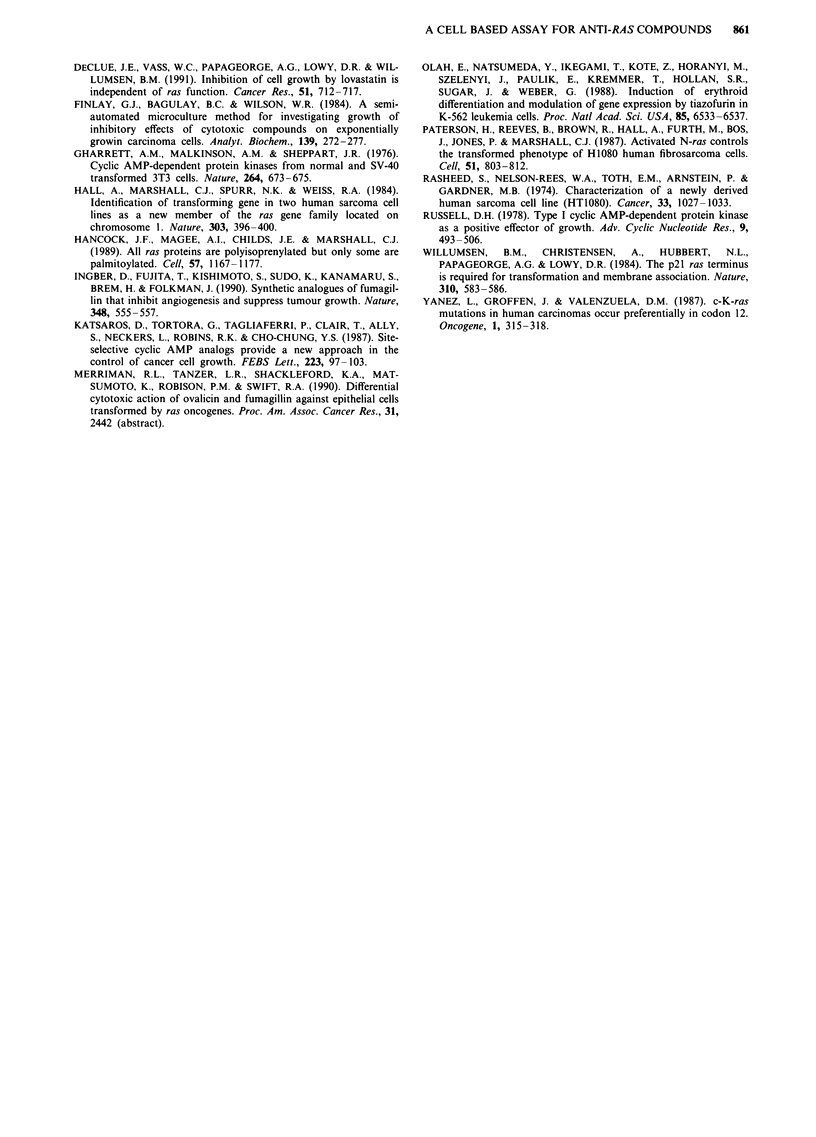# A novel cell-based assay for the evaluation of anti-ras compounds.

**DOI:** 10.1038/bjc.1993.445

**Published:** 1993-11

**Authors:** D. C. Jenkins, J. N. Stables, J. Wilkinson, P. Topley, L. S. Holmes, D. J. Linstead, E. B. Rapson

**Affiliations:** Department of Cell Biology, Wellcome Research Laboratories, Beckenham, Kent, UK.

## Abstract

**Images:**


*Br. J. Cancer* (1993), **68**, 856–861 © Macmillan Press Ltd., 1993

### A novel cell-based assay for the evaluation of anti-*ras* compounds

D.C. Jenkins, J.N. Stables, J. Wilkinson, P. Topley, L.S. Holmes, D.J. Linstead & E.B. Rapson

*Department of Cell Biology, Wellcome Research Laboratories, Beckenham, Kent BR3 3BS, UK.*

**Summary** In order to identify drugs active against mutated *ras* oncogenes we have developed an *in vitro* assay employing two clones of the human fibrosarcoma cell-line, HT1080 which carries an N-*ras* gene mutated at codon 61. Clone, HT1080scc2, retains the transformed phenotype of the parental line, whilst the other, HT1081c, is a morphologically flat, non-tumourigenic, revertant with under-representation of the chromosome carrying the transforming N-*ras* allele. The clear implication of mutant *ras* in maintaining the transformed nature of HT1080scc2 was confirmed when these cells were microinjected with the pan ras neutralising antibody Y13-259, which resulted in the morphological detransformation of these cells to a phenotype resembling that of the HT10801c clone. A number of known anti-cancer drugs with modes of action unrelated to *ras* function were found to be equipotent against both clones. However, when compounds chosen on the grounds of their potential selective cytotoxic or differentiating activity were tested some interesting results were obtained. Thus 8-bromo cAMP affected some morphological detransformation of HT1080scc2 cells and reduced their colony forming potential. The IMP-dehydrogenase inhibitors, tiazafurin and mycophenolic acid also flattened the morphology of the transformed clone. Fumagillin, an antibiotic reported to exhibit selective activity against *ras* transformed cells showed very marked and selective cytostatic effects against HT1080scc2 cells with $IC_{50}$ values as low as $1 \times 10^{-11}$M.

Mutated *ras* genes are associated with a wide variety of human tumours (Bos, 1988). Chemical modulation of the protein products of these oncogenes may lead to the detransformation of such tumours and possibly represent useful targets for selective anti-cancer chemotherapy. To test this supposition, compounds that have a modulatory effect on oncoprotein function need to be identified either through rational drug design based perhaps on the protein's structure or by the more random evaluation of compounds of diverse chemical type for leads with such activity. Both approaches required biological assays capable of detecting this *ras*-related detransformation at the cellular level.

Tumour cells that would appear to suit this purpose are the transformed sub-line, HT1080-scc2 and the revertant sub-line, HT108001c both derived (Paterson *et al.*, 1987) from the human fibrosarcoma cell line, HT1080 (Rasheed *et al.*, 1974). It has been established (Hall *et al.*, 1983; Brown *et al.*, 1984) that the parental HT1080 line contains an N-*ras* gene mutated (Gln–lys) at codon 61 and by Paterson *et al.* (1987) that in HT1080scc2, the transformed phenotype is controlled by the increased dosage of this gene relative to that found in HT10801c. Such differences in phenotype could be abolished by transfection of HT10801c with the N-*ras* oncogene from HT1080 resulting in retransformation to the HT1080scc2 phenotype. Two distinct cell sublines of the same lineage would therefore appear to depend only on their relative mutated-*ras*-gene dosage for maintenance of these differences. We reasoned that such cells might also be responsive to other modulators of *ras* function and on this basis the assay described in the present work was developed and evaluated.

### Materials and methods

### *Cell lines and culture conditions*

Transformed and revertant HT1080 sub-lines. HT1080scc2 and HT10801c were obtained from the Institute of Cancer Research, Chester Beatty Laboratories, Fulham Road, London. They were maintained routinely in Dulbecco's Modified Eagle's Medium (DMEM) supplemented with 10% foetal calf serum (FCS) and 1% penicillin/streptomycin solution containing 10,000 units per ml. All reagents were obtained from Gibco Ltd.

Cells were incubated in tissue culture grade plastic vessels at 37°C in 5% $CO_2$ in air.

### *Kinetic and morphological studies*

For determining the relative growth rates of the two lines, cells were plated out in 10 ml complete medium into 25 $cm^2$ tissue culture flasks at a density of $1 \times 10^4$ cells per ml. On each day for 6 days thereafter cells in three flasks of each line were trypsinised and the resultant cell suspensions resuspended in complete medium and counted in a Coulter counter. Four aliquots from each flask were always counted three times. The mean number of cells per ml in each group of three flasks was then calculated for each cell line and compared.

For morphological studies, flasks containing cells at pre- and post-confluent stages of growth were taken. The cells were then either examined directly by phase contrast microscopy or fixed in 3% neutral buffered formalin and stained either with Giemsa or Crystal Violet prior to microscopical examination.

To study the colony forming ability of cells under anchorage independent conditions, cells were grown in 60 mm diameter Petri dishes in 1.5 ml complete culture medium containing 0.3% agar overlaying a solid 5 ml layer of 1.8% agar. The cell concentration at the start of each experiment was $1 \times 10^5$ cells per dish. The cultures were incubated at 37°C in 5% $CO_2$ in air for 14 days and each was fed with an additional 1.5 ml of sloppy agar in complete medium on day 7. On day 13, 1 ml of 1% p-iodonitrotetrazolium violet (INT) was added to each dish. They were kept at 37°C for a further 24 h prior to examination for colony formation on day 14.

### *Microinjection experiments*

HT1080scc2 cells grown to sub-confluence on small (35 mm diameter) Petri dishes were injected intracytoplasmically either with the pan-anti-ras antibody, Y13-259 (Oncogene Science) dissolved in phosphate buffered saline to a concentration of 10 mg $ml^{-1}$ or with a similar concentration of rat $IgG_1$ control antibody, once daily for 2 days. On day 3 the test and control cells were examined microscopically for any change in morphology and their proliferative status was determined using the bromodeoxyuridine-based, Amersham Cell Proliferation Kit.

Correspondence: D.C. Jenkins.
Received 23 March 1993; and in revised form 21 June 1993.

### *Compounds tested*

The standard anti-cancer drugs tested were, doxorubicin, cisplatin, 5-fluorouracil, melphalan, 6-mercaptopurine, methotrexate and vincristine. Compounds tested on the grounds of their possible modulatory effects on cell differentiation and/or on *ras* function were, 8-bromo cAMP, mycophenolic acid, tiazofurin, mevinolin and fumagillin. Tiazofurin was obtained from Parke Davis Pharmaceuticals, Ann Arbor, Michigan, USA, mevinolin from Merck, Sharp and Dohme, Rahway, New Jersey, USA and fumagillin from Chinoin Pharmaceuticals, Hungary. All other compounds came from Sigma Chemical Company Ltd., Poole, Dorset, England.

### *Assays for drug activity*

Assays for cell proliferation/cytotoxicity were carried out in tissue culture grade 96-well microtitre plates (Costar Ltd.) Cells in log growth were added to the plates at a concentration of $1 \times 10^3$ cells per well on day 0 and serially diluted drugs were then added on day 1. Plates were then incubated at 37°C in 5% $CO_2$ in air for a further 4 days.

For quantitation of cell growth, the methylene blue biomass staining method of Finlay *et al.* (1984) was used, the test being read on a Multiscan plate reader at a wavelength of 620 nm. The morphology of the cells was checked microscopically under phase-contrast immediately before fixation and staining with methylene blue, and by ordinary light microscopy thereafter. $IC_{50}$ values for active compounds were obtained using the computer programme, GS1 and dose-response slopes were also plotted.

Where compounds were tested for activity in a colony forming assay the methods used were identical to those described earlier except that serially diluted drug was added to the sloppy agar when the test was set up, and replenished at the same concentration on day 7. The results were read on day 14.

### Results

### *Comparative growth and morphology of HT1080scc2 and HT10801c*

Growth rates in terms of cell number were similar for both lines to day 4 but thereafter HT1080scc2 cells continued to divide to reach saturation densities approximately 2 to 3 times higher than HT10801c by day 5 (Figure 1).

**Figure 1** Comparative growth of HT1080scc2 and the revertant clone, HT10801c over a 6 day time-course. Both clones were seeded at a density of $1 \times 10^4$ cells per ml on day 0.

Phenotypic differences between the two lines were clearly evident. HT10801c cells displayed a much flatter morphology then the transformed cells and only few mitotic cells were seen in confluent areas of the cultures. HT1080scc2 cells however continued to divide with numerous mitotic cells visible after confluence (Figure 2).

Grown under anchorage independent conditions in soft agar, HT1080scc2 produced several large colonies whereas HT10801c cells failed to produce any colonies greater than 0.1 mm in diameter (Figure 3).

### *Effects of microinjection with Y13-259*

HT1080scc2 cells microinjected with Y13-259 (Figure 4) developed a detransformed morphology similar to that of sub-confluent HT10801c cells. No such alteration in morphology was seen in those cells microinjected with control rat IgG. Little or no incorporation of bromodeoxyuridine was seen within the nuclei of cells injected with Y13-259 whilst those in cells injected with the control antibody were darkly stained indicative of actively dividing cells.

### *Effects of known anti-cancer drugs on growth and morphology*

IC50 values for inhibition of growth obtained for each drug against both cell lines are given in Table I. All compounds were active against both lines with $IC_{50}$ values ranging between less than $1 \times 10^{-9}$ M (vincristine) to $1 \times 10^{-5}$ M (melphalan). Individual compounds gave similar $IC_{50}$ values

**Figure 2** Comparative morphology of **a**, HT1080scc2 and **b**, HT10801c after 6 days in culture. Note the contact inhibited flatter morphology of HT10801c relative to the smaller HT1080scc2 cells which continue to divide, as evidenced by the large number of raised refractile cells still present on day 6. Bar = 30 microns.

**Figure 3** Colony growth of **a**, HT1080scc2 and **b**, HT10801c after 14 days under anchorage independent conditions in soft agar. Both clones were seeded at an initial density of 1 × $10^5$ cells per 60 mm dish on day 0. Colonies were stained with INT on day 13. Note the presence of numerous colonies of HT1080scc2 relative to the very few smaller colonies of HT10801c cells.

**Figure 4** Effects of cytoplasmic microinjection of monoclonal antibody Y13-259 on HT1080scc2 cells. Cells in **a**, were injected with Y13-259 on days 1 and 2 whilst those in **b**, were injected with rat IgG1 control antibody. All cells were stained with the bromodeoxyuridine based Amersham Cell Proliferation Kit on day 3. Note that cells in **a**, possess a flat HT10801c-like revertant morphology and that few nuclei are stained. Cells in **b**, however have retained the transformed morphology of HT1080scc2 and most nuclei are deeply stained, indicative of actively dividing cells. Bar = 10 microns.

against both lines. No obvious change in the transformed morphology of the HT1080scc2 cells towards that of the revertant HT10801c cells was seen at near- or non-toxic concentrations of any of the drugs.

*Effects of selected compounds considered likely to affect* ras *function*

The compounds, 8-bromo cAMP, mycophenolic acid, tiazofurin, mevinolin and fumagillin were evaluated against both lines (Table I). 8-bromo cAMP showed only weak cytotoxicity against either line but at concentrations down to about 5 × $10^{-5}$ M the compound did exert a morphological effect on the transformed HT1080scc2 line (Figure 5). At such concentrations flattening of the cells was seen with reversion of the transformed phenotype towards the revertant phenotype of HT10801c. This was associated with cytoplasmic spreading, restoration of signs of contact inhibition, and a marked hypochromicity to Glemsa and crystal violet staining. At a concentrtation of 1 × $10^{-4}$ M, 8-bromo cAMP also inhibited the ability of HT1080scc2 to form colonies in soft agar. Cells within the agar remained viable as determined by tetrazolium dye reduction but were prevented from growth into colonies.

The IMP dehydrogenase inhibitor, mycophenolic acid was cytotoxic against both lines with $IC_{50}$ values of 7.6 and 5.4 × $10^{-7}$ M against HT10801c and HT1080scc2 respectively. Some flattening of the scc2 cells was seen but only at concentrations where amarked degree of cytotoxicity was also evident. Tiazofurin showed similar flattening effects on the cells but was significantly less toxic with $IC_{50}$ values of 1 × $10^{-4}$ M and 5 × $10^{-5}$ M for HT10801c and HT1080scc2 respectively.

Mevinolin had no discernible detransforming effects on HT1080scc2 cells, but was cytotoxic against both these and the HT10801c cells with $IC_{50}$ values around 4 × $10^{-6}$ M for both lines.

The most pronounced differential activity was seen with the antibiotic, fumagillin, $IC_{50}$ values obtained against the revertant HT10801c were in the region of 8 × $10^{-5}$ M but against the transformed HT1080scc2 line up to 50% growth inhibition was seen at concentrations as low as 5 × $10^{-11}$ M. Dose response curves obtained for fumagillin tended to be biphasic and this phenomenon was particularly evident in one of the three repeated assays carried out on this compound (Figure 6). At concentrations ranging in four fold decrements from 8 × $10^{-4}$ M to 5 × $10^{-5}$ M the cytotoxic dose response was a steep one, but thereafter the slope was very shallow suggestive of a sustained cytostatic effect down to extremely low concentrations of the compound. In all three experiments morphology of surviving cells of neither line was affected by this drug.

### Discussion

In order to detect the effects of compounds capable of down-regulating the transforming effects of mutated ras p21 at the cellular level, *in vitro* assays employing phenotypically stable cell lines that remain responsive to ras modulation are required. Such lines should show readily discernible phenotypic differences between their transformed and their immortalised or primary states and such differences should be quantifiable. Preferably, the cell lines used should also be of human origin. Growth characteristics and morphology of HT1080scc2 were consistent with the malignant phenotype in that the cells continued to divide after confluence, grew into multiple layers on plastic and also formed colonies in soft agar. HT10801c in very obvious contrast displayed contact inhibition in culture, a much flatter morphology with few mitotic cells seen after confluence and on inability to form

**Table I** Activity of some known anti-cancer drugs and some other selected compound against HT10801c and HT1080scc2 *in vitro* (whilst $IC_{50}$ values quoted at each are from a first experiment, each was repeated at least twice giving results that did not vary significantly from the first test i.e. not by more than plus or minus one standard ×4 dilution from the original value)

| *Compound* | *$IC_{50}$(M) HT10801c* | *$IC_{50}$(M) HT1080scc2* |
|---|---|---|
| *Standard cytotoxics* | | |
| Vincristine | $<1.0\times10^{-9}$ | $<1.0\times10^{-9}$ |
| Doxorubicin | $2.2\times10^{-9}$ | $1.8\times10^{-9}$ |
| Methotrexate | $3.1\times10^{-8}$ | $1.1\times10^{-8}$ |
| 6-Mercaptopurine | $1.2\times10^{-6}$ | $1.1\times10^{-6}$ |
| 5-Fluorouracil | $2.1\times10^{-6}$ | $1.3\times10^{-6}$ |
| Cisplatin | $5.8\times10^{-6}$ | $3.4\times10^{-6}$ |
| Melphalan | $1.2\times10^{-5}$ | $1.0\times10^{-5}$ |
| *Compound with possible effects on* ras *function* | | |
| Fumagillin | $3.1\times10^{-7}$ | $1.6\times10^{-11}$ |
| Mycophenolic acid | $7.6\times10^{-7}$ | $5.4\times10^{-7a}$ |
| Mevinolin | $4.1\times10^{-6}$ | $4.3\times10^{-6}$ |
| 8-Bromo cAMP | $8.0\times10^{-5}$ | $4.8\times10^{-5a}$ |
| Tiazafurin | $1.6\times10^{-4}$ | $6.3\times10^{-5a}$ |

[a]Cells flattened and more contact inhibited indicative of morphological reversion towards HT10801c phenotype.

**Figure 6** Effects of fumagillin on growth of HT1080scc2 and HT10801c clones. Note the marked cytostatic effects of the compound on HT1080scc2 cells relative to its effects on the revertant HT10801c cells.

**Figure 5** Effects of 8-bromo cAMP on the morphology of HT1080scc2 and HT10801c clones. Cells in **a**, and **b**, are from untreated HT1080scc2 and HT10801c clones respectively whilst those in **c**, and **d**, respectively are HT1080scc2 and HT10801c exposed to 0.1 millimolar 8-bromo cAMP for 4 days. Note that in **c**, cells have developed a phenotype similar to that of **b**, and **d**, where the cells have a much more flattened morphology associated with cytoplasmic spreading and marked contact inhibition. Bar = 10 microns.

colonies under anchorage independent conditions. Such clear differences between sublines were similar to those reported previously by Paterson *et al.*, 1987 and were sufficiently consistent to lend these lines for use in drug assays based on comparative morphology and cytotoxicity. Further, the microinjection experiments described in the present work demonstrated clearly that the malignant phenotype of the HT1080scc2 cells was dependent on *ras* expression and could be modulated to the detransformed phenotype on neutralisation of the oncoprotein by the ras-specific Y13-259 antibody.

The susceptibility of both lines to known anti-cancer drugs was remarkably similar and probably reflects their common lineage. Such findings we consider important since it is well established that human tumour cell lines of analagous but not of homologous origin often differ greatly from one another with regard to their response to known anti-cancer drugs. It would indeed be potentially misleading and confusing to use cells with such widely different drug-responses as the test and control lines for identifying specific anti-ras drugs. No selective toxicity was seen against either line and none of the drugs induced any discernible detransforming effects on the HT1080scc2 cells. This itself was not surprising since these drugs are neither thought to modulate *ras* oncogenes nor to induce differentiation by any other mechanism.

Differential expression of type I and type II protein kinase isoenzymes has been shown to affect cell growth and differentiation (Gharret *et al.*, 1976; Russell, 1978; Cho-Chung, 1980) and certain analogues of c-AMP including 8-bromo cAMP have been shown to modulate this differential expression in a spectrum of cancer cell lines (Katsaros *et al.*, 1987; Ally *et al.*, 1989). The latter authors also showed that treatment with the compound decreased both N-*ras* and c-*myc* mRNA levels in lung tumours. Such observations are compatable with the present results where 8-bromo cAMP had a distinct and discernible detransforming effect on the HT1080scc2 cells consistent with the restoration of normal gene regulation in a *ras* oncogene driven tumour cell-line in which cAMP receptor proteins play a role in proliferation.

Inhibitors of IMP-dehydrogenase deplete guanylate levels within cells thereby eliciting a wide variety of intracellular effects including the down-regulation of *ras* oncogene expression (Olah *et al.*, 1988). The two IMP-dehydrodgenase inhibitors, mycophenolic acid and tiazafurin both flattened the morphology of HT1080scc2 cells and it is speculated that these effects may have been the result of *ras* downregulation. Neither compound however was effective at non-cytotoxic concentrations.

The post-translational farnesylation of ras proteins is an essential prequisite for their translocation to cell membranes (Casey *et al.*, 1989) and the inhibition of this process through the inhibition of HMG-CoA reductase, a key rate-limiting enzyme in the mevalonate pathway, should result in the depletion of membrane-associated p21. In our hands, one such inhibitor, mevinolin, was toxic to both the transformed and the revertant lines with no discernible detransforming effects on HT1080scc2. Clearly inhibition of HMG-CoA reductase could well be toxic to cells since the enzyme lies far upstream to the protein farnesylation step. The sterol biosynthetic pathway is critical for numerous cellular processes and the cytotoxicity seen in our experiments may have been unrelated to the post-translational modification of ras proteins. Noteworthy in this context are the recent observations of DeClue *et al.* (1991) who found that mevinolin was as toxic to cells transformed by v-*src* or v-*raf* as it was against cells transformed by v- or c-*ras*.

The extreme activity of fumagillin against HT1080scc2 cells deserves further investigation. This compound has anti-tumour activity which has been associated with its anti-angiogenic activity (Ingber *et al.*, 1990) and it also has been claimed (Merrimen *et al.*, 1990) to be selectively cytotoxic in a clonogenic assay against mouse mammary epithelial cells transformed by K- and H-*ras* oncogenes. The potency of the compound however was not as great as that displayed against HT1080scc2 and, at least in our assays, the compound appeared to be cytostatic rather than toxic, at a very broad range of concentrations. Clearly the cytostatic effects seen by us cannot in any way be directly associated with the compound's known anti-angiogenic activity, and at present we have no proof that the compound modulates *ras* oncogene function.

More information is required, with regard to the response of these two HT1080 sublines to a wider range of experimental compounds, before valid conclusions can be drawn as to their usefulness for evaluating compounds for anti-ras activity. We recognise that unlike HT1080, the majority of ras-associated human tumours of clinical importance are adenocarcinomas of epithelial origin and that they usually express K- rather than N-*ras* oncoproteins. Further, they usually carry mutations at codon 12 or 13 rather than at 61 (Yanez *et al.*, 1987). The significance of these differences is not known. A marked degree of homology exists between K-, N- and H-*ras* gene products (Barbacid, 1987) and we consider that compounds designed to bind to their nucleotide binding domains and modulate their function, are unlikely to prove specific for any particular *ras* sub-type. However, it is possible that compounds which interfere with the post-translational modification of ras proteins might show selectivity for one *ras* sub-type relative to another. Such, for example, could be anticipated if a compound interfered with the palmitoylation of p21. H- and N-*ras* proteins require palmitoylation prior to their translocation to the cell membrane (willumsen *et al.*, 1984; Buss & Sefton, 1986) but K-*ras* protein on the other hand does not (Hancock *et al.*, 1989). Despite such possible shortcomings, the HT1080scc2 and HT10801c clones offer an unique opportunity for the screening of compounds for anti-*ras* activity against a human tumour cell line where the test clone's malignancy has been shown to be dependent on the expression of mutant ras protein, and the non-tumourigenic control clone with its markedly different phenotype is of the same lineage.

We thank Dr Hugh Paterson of the Institute for Cancer Research, Chester Beatty Laboratories, Fulham Road, London SW3 6JB for provision of the HT1080 clones and for invaluable advice on cell microinjection techniques.

### References

ALLY, S., CLAIR, T., KATSAROS, D., TORTORA, G., YOKOZAKI, H., FINCH, R.A., AVERY, T.L. & CHO-CHUNG, Y.S. (1989). Inhibition of growth and modulation of gene expression in human lung carcinoma in athymic nude mice by site-selective 8-Cl-cyclic adenosine monophosphate. *Cancer Res.*, **49**, 5650–5655.

BARBACID, M. (1987). Ras genes. *Annu. Rev. Biochem.*, **56**, 779–828.

BOS, J.L. (1988). The *ras*-gene family and human carcinogenesis. *Mutation Res.*, **195**, 255–271.

BROWN, R., MARSHALL, C.J., PENNIE, S.G. & HALL, A. (1984). Mechanism of activation of an N-*ras* gene in the human fibrosarcoma cell line HT1080. *EMBO J.*, **3**, 1321–1326.

BUSS, J.E. & SEFTON, B.M. (1986). Direct identification of palmitic acid as the lipid attached to p21 ras. *Moll. Cell. Biol.*, **6**, 116–122.

CASEY, P.J., SOLSKI, P.A., DER, C.J. & BUSS, J.E. (1989). p21 *ras* is modified by a farnesyl isoprenoid. *Proc. Natl Acad Sci USA*, **86**, 8323–8327.

CHO-CHUNG, Y.S. (1980). Hypothesis, Cyclic AMP and its receptor protein in tumour growth regulation *in vivo*. *J. Cyclic Nucleotide Res.*, **6**, 163–177.

DECLUE, J.E., VASS, W.C., PAPAGEORGE, A.G., LOWY, D.R. & WILLUMSEN, B.M. (1991). Inhibition of cell growth by lovastatin is independent of *ras* function. *Cancer Res.*, **51**, 712-717.

FINLAY, G.J., BAGULAY, B.C. & WILSON, W.R. (1984). A semi-automated microculture method for investigating growth of inhibitory effects of cytotoxic compounds on exponentially growin carcinoma cells. *Analyt. Biochem.*, **139**, 272-277.

GHARRETT, A.M., MALKINSON, A.M. & SHEPPART, J.R. (1976). Cyclic AMP-dependent protein kinases from normal and SV-40 transformed 3T3 cells. *Nature*, **264**, 673-675.

HALL, A., MARSHALL, C.J., SPURR, N.K. & WEISS, R.A. (1984). Identification of transforming gene in two human sarcoma cell lines as a new member of the *ras* gene family located on chromosome 1. *Nature*, **303**, 396-400.

HANCOCK, J.F., MAGEE, A.I., CHILDS, J.E. & MARSHALL, C.J. (1989). All *ras* proteins are polyisoprenylated but only some are palmitoylated. *Cell*, **57**, 1167-1177.

INGBER, D., FUJITA, T., KISHIMOTO, S., SUDO, K., KANAMARU, S., BREM, H. & FOLKMAN, J. (1990). Synthetic analogues of fumagillin that inhibit angiogenesis and suppress tumour growth. *Nature*, **348**, 555-557.

KATSAROS, D., TORTORA, G., TAGLIAFERRI, P., CLAIR, T., ALLY, S., NECKERS, L., ROBINS, R.K. & CHO-CHUNG, Y.S. (1987). Site-selective cyclic AMP analogs provide a new approach in the control of cancer cell growth. *FEBS Lett.*, **223**, 97-103.

MERRIMAN, R.L., TANZER, L.R., SHACKLEFORD, K.A., MATSUMOTO, K., ROBISON, P.M. & SWIFT, R.A. (1990). Differential cytotoxic action of ovalicin and fumagillin against epithelial cells transformed by *ras* oncogenes. *Proc. Am. Assoc. Cancer Res.*, **31**, 2442 (abstract).

OLAH, E., NATSUMEDA, Y., IKEGAMI, T., KOTE, Z., HORANYI, M., SZELENYI, J., PAULIK, E., KREMMER, T., HOLLAN, S.R., SUGAR, J. & WEBER, G. (1988). Induction of erythroid differentiation and modulation of gene expression by tiazofurin in K-562 leukemia cells. *Proc. Natl Acad. Sci. USA*, **85**, 6533-6537.

PATERSON, H., REEVES, B., BROWN, R., HALL, A., FURTH, M., BOS, J., JONES, P. & MARSHALL, C.J. (1987). Activated N-*ras* controls the transformed phenotype of H1080 human fibrosarcoma cells. *Cell*, **51**, 803-812.

RASHEED, S., NELSON-REES, W.A., TOTH, E.M., ARNSTEIN, P. & GARDNER, M.B. (1974). Characterization of a newly derived human sarcoma cell line (HT1080). *Cancer*, **33**, 1027-1033.

RUSSELL, D.H. (1978). Type I cyclic AMP-dependent protein kinase as a positive effector of growth. *Adv. Cyclic Nucleotide Res.*, **9**, 493-506.

WILLUMSEN, B.M., CHRISTENSEN, A., HUBBERT, N.L., PAPAGEORGE, A.G. & LOWY, D.R. (1984). The p21 *ras* terminus is required for transformation and membrane association. *Nature*, **310**, 583-586.

YANEZ, L., GROFFEN, J. & VALENZUELA, D.M. (1987). c-K-*ras* mutations in human carcinomas occur preferentially in codon 12. *Oncogene*, **1**, 315-318.